# Prospective Evaluation of ESBL Risk Factors and Appropriateness of Empirical Therapy in Hospitalized Patients with Community-Onset Pyelonephritis

**DOI:** 10.3390/antibiotics15020229

**Published:** 2026-02-20

**Authors:** Gülşah Gelişigüzel, Şerife Altun Demircan, Murat Aysin, Esra Kaya Kılıç, Serap Yağcı, Sami Kınıklı, Rukiye Berkem

**Affiliations:** 1 Infectious Disese & Clinical Microbiology Clinic, Ankara Training and Research Hospital, Ankara 06230, Türkiye; 2 Infectious Disease & Clinical Microbiology Clinic, Ankara Etlik City Hospital, Ankara 06170, Türkiye; 3 Department of Public Health, Balıkesir University Faculty of Medicine, Balıkesir 10145, Türkiye; 4 Medical Microbiology Clinic, Ankara Training and Research Hospital, Ankara 06230, Türkiye

**Keywords:** pyelonephritis, extended spectrum beta-lactamases, antibiotic resistance, risk factors, empirical therapy

## Abstract

**Background/Objectives**: The rising prevalence of extended-spectrum beta-lactamase (ESBL)-producing pathogens has emerged as a significant challenge in the treatment of pyelonephritis. This study aims to determine the frequency of ESBL-producing agents in hospitalized patients with pyelonephritis, identify associated risk factors, and assess the appropriateness of empirical antimicrobial therapy. **Methods**: This prospective study included patients hospitalized with pyelonephritis in the Infectious Diseases Clinic of Ankara Training and Research Hospital between 1 October 2022 and 29 February 2024. Demographic features, comorbidities, urinary system pathologies, history of urinary tract interventions, hospitalization more than one month prior, antibiotic use within the previous three months, and prior urinary tract infections were compared between patients infected with ESBL-producing and non-ESBL-producing organisms. Antimicrobial susceptibility profiles and the appropriateness of empirical treatments were evaluated. Statistical analyses were performed using SPSS version 25.0, with *p* < 0.05 considered statistically significant. **Results**: *Escherichia coli* (n = 142) and *Klebsiella* spp. (n = 43) were isolated in 180 of 204 patients. ESBL positivity was detected in 95 patients (52.7%). In the multivariate logistic regression analysis, male sex (*p* = 0.038) and hospitalization more than one month prior (*p* = 0.016) were identified as independent risk factors for ESBL positivity, while prior antibiotic use in the last three months showed a borderline association (*p* = 0.055) and did not reach statistical significance. ESBL production was not associated with prolonged hospitalization; however, bacteremia significantly increased length of stay (*p* < 0.001). Antimicrobial susceptibility rates were markedly lower in the ESBL-positive group. The appropriateness of empirical therapy was also significantly reduced, with piperacillin–tazobactam being the most frequently inappropriate agent due to high resistance rates and unnecessary broad-spectrum use. **Conclusions**: ESBL-producing pathogens were highly prevalent among hospitalized patients with pyelonephritis. The low appropriateness of empirical therapy in ESBL-positive cases underscores the need for careful evaluation of ESBL risk factors prior to treatment initiation, as ESBL rates may approach 50%.

## 1. Introduction

Pyelonephritis is a complicated upper urinary tract infection and represents a major cause of community-acquired infections for which timely and appropriate antimicrobial therapy is essential [1,2]. The causative pathogens are predominantly members of the Enterobacterales family, with *Escherichia coli* being the most frequently isolated organism, followed by *Enterococcus* species [3,4,5].

Resistance to third-generation cephalosporins and other antimicrobial agents among *E. coli* and *Klebsiella* spp. has increased substantially, with a marked global rise over the past two decades [5,6]. In Türkiye, *E. coli* remains the most frequently isolated pathogen in community-acquired urinary tract infections, followed by *Klebsiella* spp., with resistance rates showing a marked increase over recent years, consistent with global trends [7]. Rising antimicrobial resistance has been linked to increased rates of complications, therapeutic failure and prolonged hospitalization [8]. Delays in initiating appropriate antibiotic therapy are associated with an increased risk of complications, including bacteremia, sepsis and acute kidney injury, and with higher morbidity and mortality [9].

In clinical practice antimicrobial therapy is frequently initiated empirically, as susceptibility results from urine cultures typically become available only after at least 48 h. Therefore, local antimicrobial resistance data play a crucial role in guiding empirical antimicrobial therapy. Although antibiotics have substantially reduced infection-related mortality, inappropriate use contributes to antimicrobial resistance, clinical failure, increased healthcare costs and *Clostridioides difficile* infection [6,10].

Although numerous studies have examined antimicrobial resistance in urinary tract infections, few have simultaneously evaluated risk factors for extended-spectrum beta-lactamase (ESBL)-producing pathogens and the appropriateness of empirical antibiotic therapy based on culture results in hospitalized patients with community-onset acute pyelonephritis. Most existing studies focus on uncomplicated urinary tract infections or heterogeneous patient populations, and prospective data from regions with high antimicrobial resistance rates remain limited [11,12].

This study aims to evaluate the distribution of causative pathogens, determine the prevalence of extended-spectrum beta-lactamase (ESBL)-producing microorganisms, identify risk factors associated with ESBL production, and assess the appropriateness of empirical antibiotic therapy in patients hospitalized with community-onset pyelonephritis.

## 2. Results

### 2.1. Study Population and Demographic Characteristics

**Figure 1:**
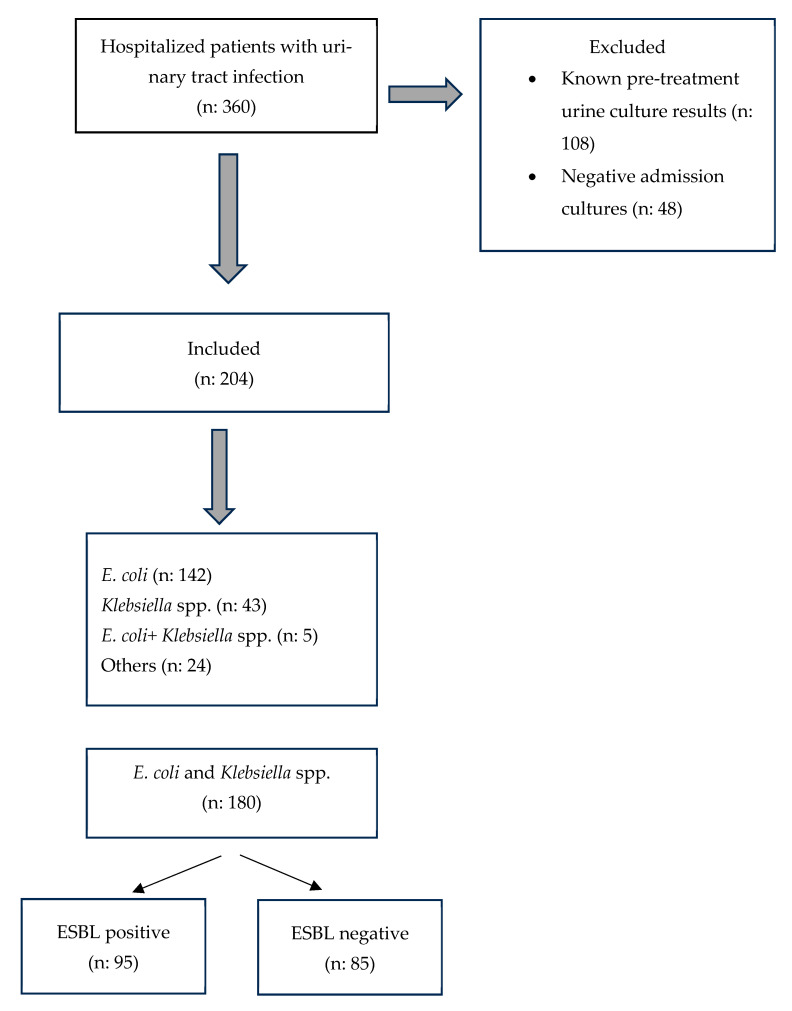
Flowchart of the proposed methodology.

A total of 360 patients hospitalized with pyelonephritis were screened during the 17-month study period. Following exclusion of 108 patients with known pre-treatment urine culture results and 48 patients with negative admission cultures, 204 patients were included in the final analysis. The flowchart of the study is presented in Figure 1.

Of these patients, 56.9% (n = 116) were female, with a mean age of 68.8 ± 18.0 years. At least one comorbidity was present in 88.7% of patients, and 26.5% (n = 54) had a history of urinary tract infection within the preceding year. Risk factors associated with pyelonephritis in the study cohort are summarized in Table 1.

Dysuria was the most common presenting symptom (67.6%), followed by chills (58.3%), fever (52.2%), nausea (52.5%), vomiting (38.7%), abdominal pain (20.1%), flank pain (17.6%), pollakiuria (14.7%), incontinence (10.8%), hematuria (8.8%), and urgency (2.9%).

### 2.2. Microbiological Results

**Figure 2:**
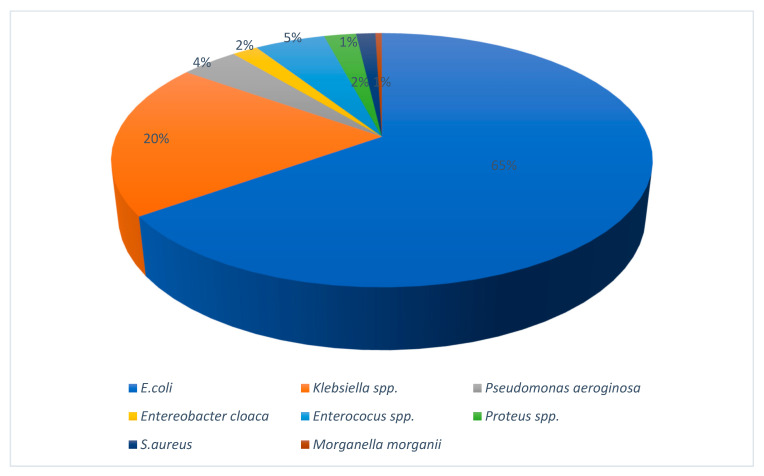
Distribution of pathogens.

A total of 218 pathogens were isolated from 204 patients. Dual pathogen growth was observed in 7% of cases. *Escherichia coli* was the most frequently isolated organism, accounting for 69.6% of all isolates. The distribution of pathogens is shown in Figure 2.

### 2.3. Distribution of ESBL and Associated Factors

Extended-spectrum beta-lactamase (ESBL)-producing organisms were identified in 95 patients, of whom 48 were male. ESBL positivity was significantly more frequent in male patients than in female patients (*p* = 0.007) (Table 2).

Among evaluated risk factors, a history of urological intervention was significantly more frequent in patients with ESBL-positive infections (*p* = 0.019) (Table 3). The mean duration of hospitalization did not differ significantly between the ESBL-positive and ESBL-negative groups (7.9 ± 2.5 vs. 7.3 ± 2.2 days; *p* = 0.078).

ESBL production rates were 52.1% in *E. coli* isolates and 55.3% in *Klebsiella* spp. isolates, with no significant difference between the two organisms (*p* = 0.73). Overall, bacteremia was observed in 24.4% of patients and in 23.2% of patients with ESBL-positive infections.

Hospitalization more than one month prior and antibiotic use within the previous three months were significantly more frequent among patients with ESBL-positive infections (*p* < 0.001 and *p* = 0.002, respectively) (Table 4).

Multivariate logistic regression identified male sex (OR = 1.85, 95% CI: 1.03–3.32; *p* = 0.038) and hospitalization more than one month prior (OR = 2.68, 95% CI: 1.20–6.00; *p* = 0.016) as independent risk factors for ESBL positivity. In contrast, prior antibiotic use showed only a borderline association and did not reach statistical significance (OR = 1.83, 95% CI: 0.99–3.37; *p* = 0.055) (Table 5).

### 2.4. Antimicrobial Susceptibility Results

Among ESBL-producing isolates, susceptibility rates were 86.8% for meropenem and amikacin, 83.5% for gentamicin and 71.4% for piperacillin–tazobactam. In contrast, susceptibility to amoxicillin–clavulanate and ciprofloxacin was substantially lower, at 35.2% and 20.2%, respectively.

Piperacillin–tazobactam resistance was identified in 26 of 91 ESBL-producing strains. Among these, 11 strains also exhibited concurrent carbapenem resistance and were excluded from further analysis. When the analysis was restricted to carbapenem-susceptible ESBL strains, the piperacillin–tazobactam resistance rate was 16.4%.

Detailed antibiotic susceptibility results are presented in Table 6.

Susceptibility rates were consistently higher among ESBL-negative isolates than among ESBL-positive isolates, with statistically significant differences observed across most antibiotics tested (*p* < 0.05). Trimethoprim–sulfamethoxazole (TMP–SMX) resistance was observed in 44.7% of ESBL-positive isolates, compared with 16.4% of ESBL-negative isolates.

Among ESBL-negative isolates, susceptibility to third-generation cephalosporins was high, with rates of 98.7% for ceftazidime, 98.7% for cefixime, and 91% for ceftriaxone. In contrast, resistance to the second-generation cephalosporin cefuroxime was observed in 50.0% of isolates.

### 2.5. Empirical Therapy and Treatment Modifications

The most frequently initiated empirical therapy was ceftriaxone (63.7%), followed by piperacillin–tazobactam (23%) and ertapenem (5.4%) (Table 7). Empirical antimicrobial therapy was modified in 53.9% of patients (n = 110), with a mean time to modification of 2.58 ± 0.87 days after treatment initiation; 89% of modifications represented escalation, while 11% represented de-escalation.

Among patients who received piperacillin–tazobactam as empirical therapy, the rate of treatment modification was significantly higher compared with those receiving other initial antibiotics (*p* = 0.036). Among the 29 patients who initially received piperacillin–tazobactam, therapy was de-escalated to ceftriaxone in 9 (31%) patients, whereas escalation was required in 20 (69%) patients. Reasons for escalation included piperacillin–tazobactam resistance (n = 11), bacteremia caused by ESBL-producing organisms (n = 4), clinical deterioration (n = 4), and concomitant soft tissue infection (n = 1).

The rate of empirical treatment modification was significantly higher in the ESBL-positive group than in the ESBL-negative group (74.7% vs. 29.4%, *p* < 0.001).

### 2.6. Clinical Outcomes and Mortality Rates

**Figure 3:**
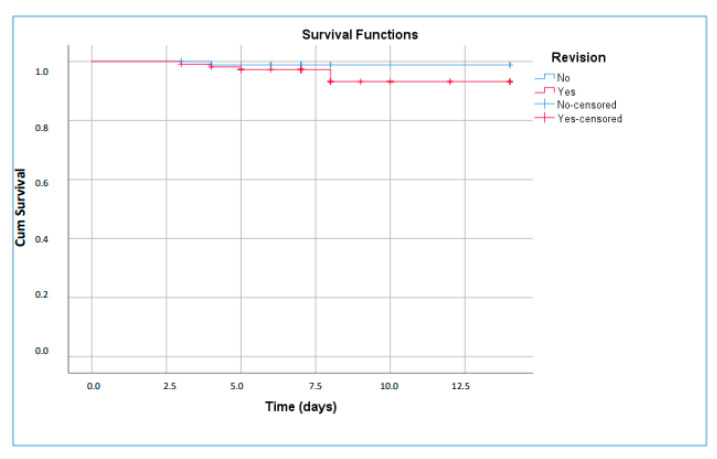
Kaplan–Meier survival curve for 30 days survival.

Of the 204 patients included in the study, 94.6% (n = 193) were discharged with clinical recovery, while 5.3% (n = 11) required admission to the intensive care unit (ICU). Eight of these patients were managed in our hospital’s ICU, and three were transferred to external centers. Among patients treated in our ICU, six died within 30 days, corresponding to an ICU mortality rate of 75%. There were no statistically significant differences in 30-day clinical outcomes between patients requiring empirical treatment modification and those who did not (Figure 3). The overall 30-day mortality rate for the entire cohort was 2.98%. Mortality data were unavailable for the three patients transferred to outside facilities.

### 2.7. Changes in Laboratory Parameters and Imaging Findings

During treatment, significant reductions were observed in white blood cell (WBC) count, C-reactive protein (CRP), procalcitonin (PCT), and creatinine levels, accompanied by a significant increase in glomerular filtration rate (GFR) (Table 8; all *p* < 0.05). The length of the hospital stay was significantly longer in patients requiring revision of empirical therapy compared with those without revision (7.9 ± 2.3 vs. 7.1 ± 2.4 days; *p* = 0.03). No significant differences were observed between the treatment-revision and non-revision groups in CRP and creatinine levels on days 0 and 3, while both groups showed significant within-group reductions by day 3 (Table 9).

Imaging was performed with ultrasonography (USG) in 126 patients, abdominal computed tomography (CT) in 40 patients, and both modalities in 21 patients. Among the 61 patients who underwent abdominal CT, 47 (77%) had imaging performed in the emergency department prior to consultation with the infectious diseases team. Among patients who underwent imaging, urolithiasis, hydronephrosis, and radiological findings consistent with pyelonephritis were identified in 13.7%, 13.7%, and 30% of cases, respectively.

## 3. Discussion

According to the Infectious Diseases Society of America (IDSA) 2010 guidelines in effect at the time the study was conducted, acute pyelonephritis is classified as either complicated or uncomplicated and all patients included in this study met the criteria for complicated pyelonephritis. In the updated IDSA 2025 guidelines, all cases of pyelonephritis are categorized as complicated urinary tract infections, reflecting an evolution in disease classification and management [1,13]. In this context, the study population may be considered broadly aligned with current definitions; however, differences in guideline frameworks over time should be taken into account when interpreting the findings.

Urinary tract infections are well established as the most common source of Gram-negative bacteremia, and timely initiation of appropriate empirical antibiotic therapy is a key determinant of clinical outcomes, including reductions in morbidity and mortality [14,15]. However, increasing antimicrobial resistance among uropathogens complicates empirical treatment selection and often necessitates broader-spectrum antibiotic use [16]. While early effective therapy remains essential, the widespread use of broad-spectrum agents accelerates the selection and dissemination of resistant strains, underscoring the need to balance prompt treatment with antimicrobial stewardship principles [17].

In our study, *E. coli* accounted for approximately 70% of isolates, and the inclusion of *Klebsiella* spp. increased this proportion to over 90%, consistent with the established literature on the etiology of urinary tract infections. The predominance of these pathogens is clinically relevant, as increasing antimicrobial resistance among community-acquired strains—both globally and in Türkiye—progressively limits empirical treatment options and complicates timely initiation of appropriate therapy [6,11,18]. Several studies from Türkiye have reported a marked increase in ESBL positivity rates over recent years and the ESBL prevalence of 52.7% observed in our cohort is consistent with recent reports involving similar hospitalized patient populations [19]. This relatively high prevalence may, at least in part, reflect the inclusion of exclusively hospitalized patients and the increasing resistance patterns observed in our setting.

Although no significant difference was observed in bacteremia rates between patients infected with ESBL-positive and ESBL-negative pathogens, bacteremia was associated with a significantly longer length of hospital stay (*p* < 0.001) [20]. Although the length of hospital stay was somewhat longer among patients with bacteremia in the ESBL-positive group compared with the ESBL-negative group, this difference did not reach statistical significance. While several studies have reported prolonged mean lengths among patients infected with ESBL-producing pathogens, our findings demonstrate comparable mean lengths of stay between the two groups [21,22]. This observation may be related to the patient profile of our center. Given the high proportion of elderly patients, individuals with low socioeconomic status, and those requiring ongoing care, completion of treatment may have been preferred in order to mitigate potential challenges related to adherence to outpatient therapy after discharge.

According to 2023 data from the WHO European Region Antimicrobial Medicines Consumption Network, Türkiye is among the countries with the highest antibiotic consumption in the European Region [23]. This observation provides important contextual information that may be relevant when interpreting the high resistance rates observed in our study.

In the present study, empirical treatment modification was significantly more frequent among patients with ESBL-producing pathogens, reflecting the challenges of selecting adequate initial therapy in settings with high antimicrobial resistance. Previous studies have consistently identified recent antibiotic exposure, recent hospitalization, invasive procedures, and urinary catheterization as important risk factors for ESBL-producing Enterobacterales infections [12,19]. Consistent with previous reports, our univariate analysis demonstrated that both hospitalization more than one month prior and antibiotic use within the previous three months were associated with ESBL positivity. However, in the multivariate model, only male sex and hospitalization more than one month prior remained independently associated with ESBL-producing infections, while prior antibiotic use showed a borderline association that did not reach statistical significance.

These associations are biologically plausible, as prior antibiotic exposure exerts selective pressure that facilitates the emergence and persistence of resistant strains, while previous hospitalization beyond one month increases exposure to healthcare-associated pathogens with higher resistance profiles. Importantly, the presence of these clinical risk indicators—particularly hospitalization more than one month prior—was associated with a higher likelihood of inadequate empirical therapy, resulting in subsequent treatment modification. Our findings reinforce the need to incorporate readily identifiable patient-specific risk factors into empirical antibiotic decision-making to improve initial treatment adequacy and potentially reduce the need for later therapeutic adjustments.

The high rate of empirical piperacillin–tazobactam failure observed in this study may be explained by the elevated local prevalence of ESBL-producing organisms. Although a local antibiogram was available, the absence of an active antimicrobial stewardship program may have limited the integration of resistance data into individualized empirical treatment decisions. These findings emphasize the importance of stewardship-supported, risk-based empirical antibiotic selection in regions with high ESBL rates. Notably, in addition to the high ESBL prevalence, a substantial proportion of patients with carbapenem-resistant isolates had identifiable healthcare-related exposures. Among the 11 patients with carbapenem-resistant Enterobacterales (CRE), 10 isolates were Klebsiella spp. and one was Escherichia coli. Five patients had a history of hospitalization more than one month prior and ten had recent antibiotic exposure; two were receiving chronic hemodialysis, and risk factors frequently overlapped. Both dialysis patients had prior hospitalization and antibiotic use. Although these infections met the criteria for community-onset based on timing, the presence of overlapping healthcare-related exposures suggests that carbapenem resistance in these cases may, at least in part, reflect healthcare-associated acquisition and cumulative antimicrobial pressure rather than true community acquisition. Patients undergoing dialysis represent a particularly vulnerable subgroup due to frequent healthcare contact and repeated antimicrobial exposure. From a clinical perspective, the presence of CRE among ESBL-producing isolates challenges the assumption that carbapenems remain universally reliable last-line agents in our setting and underscores the importance of careful risk stratification and consideration of local epidemiological data when selecting empirical therapy. This observation is consistent with previous reports indicating that community-onset CRE infections are often linked to prior healthcare exposure, including recent hospitalization, dialysis, and cumulative antibiotic use [24].

From an antimicrobial stewardship perspective, broad-spectrum antibiotic use is a well-recognized risk factor for the development of antibiotic resistance, as excessive or unnecessary exposure exerts selective pressure on microbial populations [25]. In the context of urinary tract infections, Alshareef and colleagues demonstrated that implementation of antibiotic de-escalation strategies not only reduced the length of hospital stay but also lowered the likelihood of multidrug-resistant pathogen emergence [26]. Consequently, initiating broad-spectrum antibiotics unnecessarily or failing to de-escalate therapy when appropriate can contribute to the selection and proliferation of resistant strains, highlighting the importance of antibiotic stewardship in clinical practice.

Routine imaging is generally not required in acute pyelonephritis; however, ultrasonography or computed tomography should be reserved for patients with delayed clinical response, suspected complications or specific risk factors [27]. In our cohort, a substantial proportion of computed tomography examinations were performed in the emergency department prior to Infectious Diseases consultation. In settings with high patient volumes and limited access to early subspecialty consultation, diagnostic imaging may be more frequently utilized, sometimes independent of strict clinical indications. While such practices may facilitate rapid decision-making, early or routine computed tomography in clinically uncomplicated cases is likely to provide limited additional diagnostic value, while exposing patients to avoidable radiation and increasing healthcare costs [28]. These findings highlight the need for more judicious, guideline-based use of imaging modalities.

This study has several limitations that should be acknowledged. First, its single-center design may limit the generalizability of the findings to other settings with different patient populations and antimicrobial resistance patterns. In addition, inclusion of only hospitalized patients may not fully represent the entire spectrum of community-onset pyelonephritis, particularly cases managed in the outpatient setting. Second, although multivariate analysis was performed, residual confounding inherent to observational studies cannot be completely excluded. Comorbidities were evaluated; however, polypharmacy was not systematically assessed and therefore could not be analyzed as a potential risk factor for ESBL production. Third, empirical antibiotic selection may have been influenced by institutional factors, including the absence of an active antimicrobial stewardship program and the limited availability of ertapenem during the study period. Furthermore, molecular characterization of ESBL-producing isolates was not performed due to technical and resource limitations, precluding detailed analysis of resistance mechanisms and clonal epidemiology. Finally, mortality outcomes could not be fully ascertained for patients transferred to external intensive care units, potentially leading to an underestimation of overall mortality.

The findings of this study suggest that empirical antibiotic therapy for hospitalized patients with pyelonephritis should be guided by local pathogen distribution and antimicrobial resistance patterns. In centers with a high prevalence of ESBL-producing organisms, the empirical use of piperacillin–tazobactam should be carefully considered, as it may necessitate frequent treatment modifications. Early revision of therapy based on culture results and the implementation of de-escalation strategies in appropriate patients should therefore be prioritized. Such an approach may support clinical success while reducing unnecessary use of broad-spectrum antibiotics and enhancing the effectiveness of antimicrobial stewardship programs.

## 4. Materials and Methods

This prospective study was conducted between 1 October 2022 and 29 February 2024 in the Infectious Diseases Department of a 670-bed tertiary care teaching and research hospital in Ankara, the capital of Türkiye. Ethical approval was obtained from the Clinical Research Ethics Committee of the University of Health Sciences Ankara Training and Research Hospital on 14 December 2022 (approval number: E-22-1100).

### 4.1. Patient Selection and Study Population

Patients aged ≥18 years who were hospitalized with a presumptive diagnosis of pyelonephritis and who received empirical antibiotic therapy were included in the study. Pyelonephritis was diagnosed based on clinical findings, including fever (≥38 °C), chills or rigors, flank pain, nausea, vomiting, and costovertebral angle tenderness. Patients presenting with urinary symptoms such as dysuria or pollakiuria in the absence of systemic signs were classified as having cystitis and were excluded from the study.

Community-onset pyelonephritis was defined as acute pyelonephritis in patients who presented from the community, with symptom onset occurring before hospital admission or within the first 48 h of hospitalization. Patients with symptoms developing after 48 h of hospitalization were excluded. Previous healthcare exposures, including hospitalization more than one month prior, urinary tract interventions, or prior urinary catheterization, were not considered exclusion criteria. Healthcare exposures occurring between 30 and 90 days prior to admission were recorded as potential risk factors but were not considered exclusion criteria.

According to the IDSA guideline dated to 17 July 2025, pyelonephritis is classified as a complicated urinary tract infection. However, at the time the study was conducted, factors such as male sex, pregnancy, urinary catheterization, anatomical or functional abnormalities (e.g., urolithiasis), and comorbid conditions including diabetes mellitus were considered complicating factors and were therefore classified as complicated pyelonephritis.

Patients whose urine culture results were already available at the time of admission and those with no growth in urine cultures obtained prior to empirical therapy were excluded from the study.

### 4.2. Data Collection

Demographic characteristics (including age and sex), urinary and systemic symptoms, comorbidities, coinfections, physical examination findings (including fever, mental status and costovertebral angle tenderness), and laboratory parameters—namely complete blood count, C-reactive protein (CRP), procalcitonin (PCT), blood urea nitrogen (BUN), creatinine, and glomerular filtration rate (GFR)—were prospectively recorded using standardized follow-up forms. Empirical antibiotic therapies and subsequent targeted treatments administered after the availability of urine culture results were also documented.

### 4.3. Sample Processing and Microbiological Evaluation

Pyuria was defined as the presence of ≥10 leukocytes per mm^3^ on microscopic examination of an uncentrifuged urine specimen using a Thoma counting chamber. For urine culture, samples were inoculated onto blood agar and Eosin Methylene Blue (EMB) agar using a calibrated 0.01 mL loop and incubated at 37 °C for 16–24 h. Following incubation, the growth of a single organism or two organisms at a concentration of ≥10^5^ colony-forming units (CFU)/mL was considered significant bacteriuria.

Identification of isolates with significant growth was performed using the VITEK^®^ MS Pioneering Diagnostics- (bioMérieux, Marcy-l’Étoile, France). Antimicrobial susceptibility testing of Gram-negative pathogens considered causative agents was performed using VITEK-2^®^ AST-N423 cards (bioMérieux, Marcy-l’Étoile, France). Antibiotic susceptibility profiles and ESBL phenotypes were determined using the VITEK-2 Compact system (bioMérieux, France). Susceptibility results were categorized as susceptible, resistant or susceptible at increased exposure based on the automated system. In parallel, antimicrobial susceptibility testing was also performed using the Kirby–Bauer disk diffusion method.

*E. coli* or *Klebsiella* spp. isolates reported as ESBL-positive by the laboratory were classified as ESBL-producing strains.

### 4.4. Antimicrobial Susceptibility Testing

Antimicrobial susceptibility profiles and ESBL production of the study isolates were additionally assessed using the disk diffusion method according to the European Committee on Antimicrobial Susceptibility Testing (EUCAST) breakpoint tables (Version 12, 2022) [29].

Disk diffusion testing was performed using antibiotic disks supplied by Bioanalyse (Türkiye), including amoxicillin–clavulanic acid (20/10 µg), aztreonam (30 µg), cefuroxime (30 µg), ceftriaxone (30 µg), ceftazidime (30 µg), cefoxitin (30 µg), piperacillin–tazobactam (100/10 µg), imipenem (10 µg), meropenem (10 µg), ciprofloxacin (5 µg), gentamicin (10 µg), fosfomycin (50 µg) and trimethoprim–sulfamethoxazole (1.25/23.75 µg).

Bacterial suspensions were adjusted to a 0.5 McFarland standard and inoculated onto Mueller–Hinton agar plates (BD, France). An amoxicillin–clavulanic acid disk was placed at the center of the agar plate, while ceftazidime, ceftriaxone, cefoxitin, aztreonam, and imipenem disks were positioned at a distance of 25 mm from the central disk for ESBL screening.

Plates were incubated at 35 °C for 18–24 h in an incubator (EN 500, NÜVE, Ankara, Turkey). Antimicrobial susceptibility results were interpreted according to EUCAST criteria.

### 4.5. Antimicrobial Therapy

All antimicrobial doses were calculated according to Sanford guidelines, considering patient-specific factors such as renal function (eGFR), body weight, and BMI.

### 4.6. Study Endpoints

The primary endpoint of the study was the prevalence of ESBL-producing pathogens among hospitalized patients with community-onset pyelonephritis. Secondary endpoints included identification of risk factors associated with ESBL production, evaluation of empirical antimicrobial therapy modification, length of hospital stay, bacteremia and ICU admission rates, 30-day mortality, and changes in laboratory parameters during treatment.

### 4.7. Statistical Analysis

Statistical analyses were performed using the Statistical Package for the Social Sciences (SPSS) for Windows, version 25.0 (IBM Corp., Armonk, NY, USA). The distribution of continuous variables was assessed using visual inspection of histograms and analytical tests, including the Kolmogorov–Smirnov and Shapiro–Wilk tests. Categorical variables were summarized as numbers and percentages, while normally distributed continuous variables were expressed as means and standard deviations. Student’s *t*-test was used to compare continuous variables between two groups, whereas analysis of variance (ANOVA) was applied for comparisons among three or more groups. Comparisons of categorical variables were performed using the chi-square test or Fisher’s exact test, as appropriate. A *p* value of <0.05 was considered statistically significant. In addition, a priori sample size and power calculation was performed using G*Power version 3.1.9. Based on a previous study with a similar design, assuming a power of 90%, an effect size of 0.3 (medium effect for chi-square test), and an alpha error of 0.05, a minimum sample size of 183 patients was required [30].

## 5. Conclusions

In summary, this prospective single-center study provides contemporary real-world data on the epidemiology of community-onset pyelonephritis requiring hospitalization in a setting with high antimicrobial resistance. The high prevalence of ESBL-producing *Enterobacterales* and the frequent need for empirical treatment modification—particularly among patients initially treated with piperacillin–tazobactam—highlight the limitations of standardized empirical regimens in regions with evolving resistance patterns. These findings reinforce the critical role of local surveillance data, early culture-guided therapy optimization and antimicrobial stewardship-oriented decision-making in improving clinical outcomes while minimizing unnecessary broad-spectrum antibiotic exposure. Collectively, these results underscore the imperative for context-specific empirical therapeutic strategies and reinforce the value of individualized, data-driven decision-making in the management of hospitalized patients with acute pyelonephritis.

## Figures and Tables

**Table 1:** Urinary-tract-related risk factor (n = 204).

**Variable**	**Yes n (%)**	**No n (%)**
History of UTI	54 (26.5)	150 (73.5)
CKD/Dialysis	27 (13.2)	177 (86.8)
Transurethral catheterization	24 (11.8)	180 (88.2)
Nephrostomy	2 (1)	202 (99)
Urethral Stent	2 (1)	202 (99)
Anatomical defect	10 (4.9)	194 (95.1)
Neurogenic bladder	11 (5.4)	193 (94.6)
Vesicoureteral reflux	2 (1)	202 (99)
BPH	46 (22.5)	158 (77.5)
Prostatitis	1 (0.5)	203 (99.5)
Urolithiasis	28 (13.7)	176 (86.3)
Urinary Intervention	31 (15.2)	174 (84.8)

UTI: urinary tract infection, CKD: chronic kidney disease.

**Table 2:** Demographic characteristics and comorbidities of patients according to ESBL status.

**Variable**	**ESBL Positive** **n (%)**	**ESBL Negative** **n (%)**	***p* Value**
Male	48 (64.9)	26 (35.1)	0.007 ^1^
Age (mean ± SD)	68.2 ± 16.7	68.7 ± 18.4	0.831 ^2^
Uncontrolled DM	42 (53.8)	36 (46.2)	0.802 ^1^
Hypertension	54 (57.4)	40 (42.6)	0.190 ^1^
Heart Failure	18 (60.0)	12 (40.0)	0.385 ^1^
COPD/Asthma	15 (57.7)	11 (42.3)	0.587 ^1^
Neurological disease	29 (52.7)	26 (47.3)	0.993 ^1^
Malignancy	11 (55.0)	9 (45.0)	0.833 ^1^
Immunosuppression	9 (69.2)	4 (30.8)	0.217 ^1^
CKD/Dialysis	15 (60.0)	10 (40.0)	0.436 ^1^

DM: diabetes mellitus, COPD: chronic obstructive pulmonary disease, CKD: chronic kidney disease, ^1^ Chi-square test, ^2^ *t*-test.

**Table 3:** ESBL distribution according to patient-related risk factors (n = 204).

**Variable**	**ESBL Positive** **n (%)**	**ESBL Negative n (%)**	***p* Value**
Transurethral Catheterization			0.455 ^1^
Yes	11 (61.1)	7 (38.9)	
No	84 (51.9)	78 (48.1)	
Nephrostomy/Urethral Stent			0.499 ^2^
Yes	2 (100.0)	0	
No	93 (52.2)	85 (47.8)	
Anatomical Defect			0.449 ^2^
Yes	5 (71.4)	2 (28.6)	
No	90 (52.0)	83 (48.0)	
Neurogenic Bladder			1.000 ^2^
Yes	5 (55.6)	4 (44.4)	
No	90 (52.6)	81 (47.4)	
Vesicoureteral reflux			0.499 ^2^
Yes	2 (100.0)	0	
No	93 (52.2)	85 (47.8)	
BPH			0.149 ^1^
Yes	24 (63.2)	14 (36.8)	
No	71 (50.0)	71 (50.0)	
Prostatitis			1.000 ^2^
Yes	1 (100.0)	0	
No	94 (52.5)	85 (47.5)	
Urolithiasis			0.175 ^1^
Yes	14 (66.7)	7 (33.3)	
No	81 (50.9)	78 (49.1)	
Uriner Intervention			0.019 ^1^
Yes	18 (75.0)	6 (25.0)	
No	77 (49.4)	79 (50.6)	
Urine Culture			0.730 ^1^
*E. coli*	74 (52.1)	68 (47.9)	
*Klebsiella* spp.	21 (55.3)	17 (44.7)	
Bacteremia			0.671 ^1^
Yes	22 (23.2)	22 (25.9)	
No	73 (76.8)	63 (74.1)	
Hospitalization more than one month prior			<0.001 ^1^
Yes	28 (80.0)	7 (20.0)	
No	67 (46.2)	78 (53.8)	
Antibiotic exposure in the previous three months			0.002 ^1^
Yes	47 (67.1)	23 (32.9)	
No	48 (43.6)	62 (56.4)	
History of urinary tract infection within the previous year			0.216 ^1^
Yes	29 (60.4)	19 (39.6)	
No	66 (50.0)	66 (50.0)	
Duration (days)			0.078 ^3^
Mean ± SD	7.9 ± 2.5	7.3 ± 2.2	

^1^ Chi-square test, ^2^ Fisher’s exact test, ^3^ *t*-test.

**Table 6:** Comparison of antibiotic susceptibility between ESBL-positive and ESBL-negative isolates.

	**ESBL Positive** **n: 91**	**ESBL Negative** **n: 78**	***p* Value**
**Amikacin**			0.001 ^1^
Resistant	5 (5.5)	0 (0)	
Sensitive to high doses	7 (7.7)	0 (0)	
Sensitive	79 (86.8)	78 (100.0)	
**Amoxicillin–clavulanic acid**			<0.001 ^1^
Resistant	59 (64.8)	9 (11.5)	
Sensitive	32 (35.2)	69 (88.5)	
**Ciprofloxacin**			<0.001 ^1^
Resistant	58 (65.2)	7 (9.0)	
Sensitive to high doses	13 (14.6)	0(0)	
Sensitive	18 (20.2)	71 (91.0)	
**Gentamicin**			0.001 ^1^
Resistant	15 (16.5)	1 (1.3)	
Sensitive	76 (83.5)	77 (98.7)	
**Meropenem**			0.001 ^1^
Resistant	10 (11.0)	0 (0)	
Sensitive to high doses	2 (2.2)	0 (0)	
Sensitive	79 (86.8)	0 (0)	
**Piperacillin–Tazobactam**			0.001 ^1^
Resistant	26 (28.6)	1 (1.3)	
Sensitive	65 (71.4)	77 (98.7)	

^1^ Chi-square test.

**Table 7:** Rate of antibiotic revisions.

**Antibiotic Change**	**n (%)**
**All treatments**	
Present	110 (53.9)
Absent	94 (46.1)
**Ceftriaxone**	
Present	70 (53.8)
Absent	60 (46.2)
**Piperacillin–Tazobactam**	
Present	29 (72.5)
Absent	11 (27.5)
**Ertapenem/Meropenem**	
Present	7 (35.0)
Absent	13 (65.0)

**Table 8:** Changes in laboratory parameters during hospitalization.

**Title 1**	**Day of Admission**	**Day 3 of Treatment**	**Day of Discharge**	***p* Value**
WBC (mean ± SD)	13.9 ± 6.2	9.1 ± 4.4	8.6 ± 3.7	<0.001 ^1^
CRP (mean ± SD)	140.7 ± 93.8	99.7 ± 76.3	29.2 ± 27.1	<0.001 ^1^
PCT (mean ± SD)	5.1 ± 9.0	4.0 ± 7.9	0.4 ± 0.4	<0.001 ^1^
Creatinin (mean ± SD)	1.5 ± 1.5	1.1 ± 0.7	1.0 ± 0.8	<0.001 ^1^
GFR (mean ± SD)	59.7 ± 30.9	71.0 ± 32.8	75.8 ± 31.5	<0.001 ^1^
Complete urinalysis	146.3 ± 73.7	17.4 ± 42.6	-	<0.001 ^2^

WBC: white blood cell, CRP: C-reactive protein, PCT: procalcitonin, GFR: glomerular filtration rate, ^1^ variant analysis, ^2^ paired samples *t*-test.

**Table 4:** Association between patient characteristics and ESBL production.

**Variable**	**ESBL Positive** **n (%)**	**ESBL Negative n (%)**	***p* Value**
Hospitalization more than one month prior			<0.001 ^1^
Yes	28 (80.0)	7 (20.0)	
No	67 (46.2)	78 (53.8)	
Antibiotic exposure in the previous three months			0.002 ^1^
Yes	47 (67.1)	23 (32.9)	
No	48 (43.6)	62 (56.4)	
History of urinary tract infection within the previous year			0.216 ^1^
Yes	29 (60.4)	19 (39.6)	
No	66 (50.0)	66 (50.0)	

^1^ Chi-square test.

**Table 5:** Univariable and multivariable analyses of the risk factors for ESBLdevelopment.

**Risk Factors**	**Univariable Analysis**		**Multivariable Analysis**	
	**Odds Ratio (95%** **Confidence Interval)**	***p* Value**	**Adjusted Odds Ratio (95% Confidence Interval)**	***p* Value**
Male	1.864 (1.064–3.266)	0.007	1.853 (1.034–3.319)	0.038
Hospitalization more than one month prior	3.455 (1.611–7.407)	0.001	2.683 (1.200–6.002)	0.016
Antibiotic exposure in the previous three months	2.220 (1.251–3.939)	0.002	1.825 (0.987–3.374)	0.055

^1^ Chi-square test.

WBC: white blood cell, CRP: C-reactive protein, PCT: procalcitonin, GFR: glomerular filtration rate, ^1^ variant analysis, ^2^ paired samples *t*-test.

**Table 9:** Comparison of CRP and creatinine levels on days 0 and 3 in the treatment-revision and non-revision groups.

**Parameter**	**Time Point**	**Treatment Revision (Yes) Mean ± SD (n = 104)**	**Treatment Revision (No) Mean ± SD (n = ** **73–75)**	***p* Value**
CRP (mg/L)	Day 0	136.6 ± 91.3	142.9 ± 92.8	0.992
CRP (mg/L)	Day 3	94.4 ± 74.0	103.8 ± 76.9	0.416
Creatinin (mg/dL)	Day 0	1.4 ± 1.7	1.5 ± 0.8	0.956
Creatinin (mg/dL)	Day 3	1.1 ± 0.7	1.0 ± 0.5	0.912

## Data Availability

The data presented in the study are available on request from the corresponding author.

## Abbreviations

The following abbreviations are used in this manuscript:

ESBL	Extended spectrum beta-lactamases
UTI	Urinary tract infection
ICU	Intensive care unit
DM	Diabetes mellitus
